# Mitral Annular Calcification: Natural History, Prognosis, and Clinical Outcomes

**DOI:** 10.1016/j.jscai.2025.103732

**Published:** 2025-07-22

**Authors:** Gustavo Mendez-Hirata, Christian W. Schmidt, Hanad I. Bashir, Kofi N. Ansah, Geoffrey A. Answini, J. Michael Smith, Saad Hasan, Jeffrey Griffin, Robert Dowling, Meghna Seshiah, Alton Headworth, Raviteja R. Guddeti, Nadia El Hangouche, Richard Bae, Puvi Seshiah, Dean J. Kereiakes, Santiago Garcia

**Affiliations:** The Christ Hospital Heart and Vascular Institute, The Lindner Center for Research and Education, Cincinnati, Ohio

**Keywords:** mitral annular calcification, mitral regurgitation, mitral stenosis, mitral valve dysfunction

## Abstract

**Background:**

Mitral annular calcification (MAC) is a chronic and progressive degenerative process characterized by calcium and lipid deposition of the mitral valve annulus. We sought to describe the natural history of patients with MAC with and without mitral valve dysfunction (MVD).

**Methods:**

We conducted an observational study of patients with an echocardiogram-based diagnosis of MAC from 2006-2023. Patients were matched by age and sex in a 1:1 ratio to patients without MAC. We collected baseline clinical, echocardiographic, and computed tomography data. The primary end point was all-cause mortality. We also report predictors of mortality in patients with MAC and MVD.

**Results:**

We included 15,372 patients in the analysis: 7686 with MAC (median age, 76 years [68-84], 58% women) and 7686 without MAC matched by age and sex. Patients with MAC had higher rates of comorbidities, cardiovascular risk factors, MVD, and multivalvular heart disease (aortic stenosis/regurgitation and tricuspid regurgitation) relative to patients without MAC (all *P* < .001). Two-year mortality was 14%, 26%, and 21% for patients with MAC and no MVD, MAC with ≥moderate MR, and MAC with ≥moderate MS, respectively (*P* < .001). Surgical or transcatheter mitral valve interventions in patients with MAC and significant MVD were infrequently performed (n = 136/788, 17.2%).

**Conclusions:**

Ten percent of patients with MAC have clinically significant MVD, with mitral regurgitation being more common than mitral stenosis. The presence of MVD is associated with significantly increased mortality in patients with MAC.

## Introduction

Mitral annular calcification (MAC) is a chronic and progressive degenerative process characterized by calcium and lipid deposition in and around the fibrous base of the mitral valve annulus. It is hypothesized to result from increased mitral valve mechanical stress, dysregulated calcium and phosphate metabolism, and increased inflammation in the mitral valve annulus.^1^^,^^2^ The true incidence of MAC is challenging to estimate, but studies suggest it ranges between 5% and 15% in the general population and increases to >40% in the elderly.^3^ MAC is associated with a higher burden of cardiovascular (CV) comorbidities and atherosclerotic risk factors.^4^, ^5^, ^6^

Mitral annular calcification, usually found on echocardiography or computed tomography, can extend into the mitral valve leaflets, leading to mitral valve dysfunction (MVD), which can present as mitral regurgitation (MR), mitral stenosis (MS), and/or mixed MVD (MR+MS). The presence of MVD in patients with MAC has been associated with increased mortality and worse outcomes.^3^ It is universally accepted that MAC patients constitute a difficult group to treat with surgical or transcatheter interventions owing to clinical and anatomical factors.^7^ In this study, we aimed to analyze clinical characteristics, prognosis, and outcomes of patients with MAC with and without MVD.

## Materials and methods

### Study design

We conducted an observational study of patients with an echocardiogram-based diagnosis of MAC at a single institution (The Christ Hospital, Cincinnati, Ohio). We used advanced analytics (Microsoft Azure Data Studio) to retrospectively query the institutional echocardiography database and electronic medical records (EPIC systems) to identify all patients with a diagnosis of MAC by transthoracic echocardiogram from 2006-2023. The diagnostic transthoracic echocardiogram was performed under the clinical judgment of the patient’s provider. In patients with multiple echocardiograms, the first study was used to confirm the diagnosis (diagnostic), while the last echocardiogram was used to examine the progression of MVD whenever available. Baseline clinical comorbidities were collected using International Classification of Diseases codes, recorded prospectively by the patient provider at each encounter. To compare baseline clinical characteristics, echocardiogram data, and outcomes, patients with MAC were matched in a 1:1 ratio by age and sex with patients receiving an echocardiogram at The Christ Hospital without a diagnosis of MAC during the same study period. Mitral valve intervention data (surgical or transcatheter) were collected only for the MAC group, and the decision to proceed with either treatment strategy was made by the treating physician. The decision not to offer treatment was also captured. We excluded patients with a history of heart transplant or congenital heart disease.

### Echocardiogram

The echocardiogram reports of all patients included in the study were retrieved and independently analyzed for data collection. Valvular (aortic, mitral, and tricuspid) regurgitation and stenosis severity were classified according to the American Society of Echocardiography recommendations.^8^, ^9^, ^10^ MVD was considered significant if it was equal or greater than moderate for both MR and MS based on existing guidelines. We evaluated extra valvular heart function by assessing left ventricular ejection fraction, left ventricular end-systolic and diastolic dimensions, left atrial volume index, right ventricular systolic pressure, tricuspid annular plane systolic excursion, and basal right ventricular basal end-diastolic dimension.

### MAC cardiac computed tomography score

We identified all patients with a gated contrasted cardiac computed tomography performed during the study period. The images were analyzed, and measurements were made using 3mensio software (Pie Medical Imaging). Mitral valve anatomy was evaluated in the best diastolic phase using 3mensio Mitral Workflow. Calcium volume was calculated visually using the calcium quantification tool and selectively isolating the calcification in the mitral valve annulus. The severity of MAC was classified following the recommendations and criteria from the MAC computed tomography score^11^ into mild (MAC score, 0-3), moderate (MAC score, 4-6), and severe (MAC score, 7-10). The MAC score takes into consideration calcium thickness, calcium distribution, trigone, and leaflet involvement. Of note, most of the cardiac computed tomography studies included were performed in preparation for transcatheter aortic valve replacement.

### Statistical analysis

Continuous variables are presented as mean ± SD if normally distributed or median (IQR) if skewed. Discrete variables are presented as counts and percentages. Differences in continuous variables were assessed using a *t* test or 1-way ANOVA if normally distributed. Nonnormally distributed continuous variables were assessed for differences using the Wilcoxon rank-sum test or the Kruskal-Wallis test. Categorical variables were assessed using the χ^2^ test or Fisher exact test, where appropriate.

The primary end point was all-cause mortality. Mortality was assessed by review of electronic medical records, and death certificates when appropriate and included both inpatient and outpatient events. The secondary outcome was CV-related mortality in patients with moderate-severe/severe MS and/or MR who did not receive an intervention (surgical or transcatheter) compared with patients who did. CV-related mortality was defined as death due to acute myocardial infarction, heart failure, stroke, CV procedures, CV hemorrhage, and sudden cardiac death. Patient survival is reported using Kaplan-Meier curves, with differences assessed using the Peto-Prentice test. Cox proportional hazard models were created, and proportional hazard assumptions were tested to assess the influence of characteristics on patient survival. All covariates were assessed using univariate models and models that adjusted for age, sex, and MR severity. All variables that were significant at *P* < .10 in the adjusted models were included for possible selection into a final model using backward stepwise selection. All analyses were performed using Stata version 17.0 (StataCorp LLC). The study was approved by the local institutional review board; informed consent was waived.

## Results

### Patient characteristics

**Figure 1:**
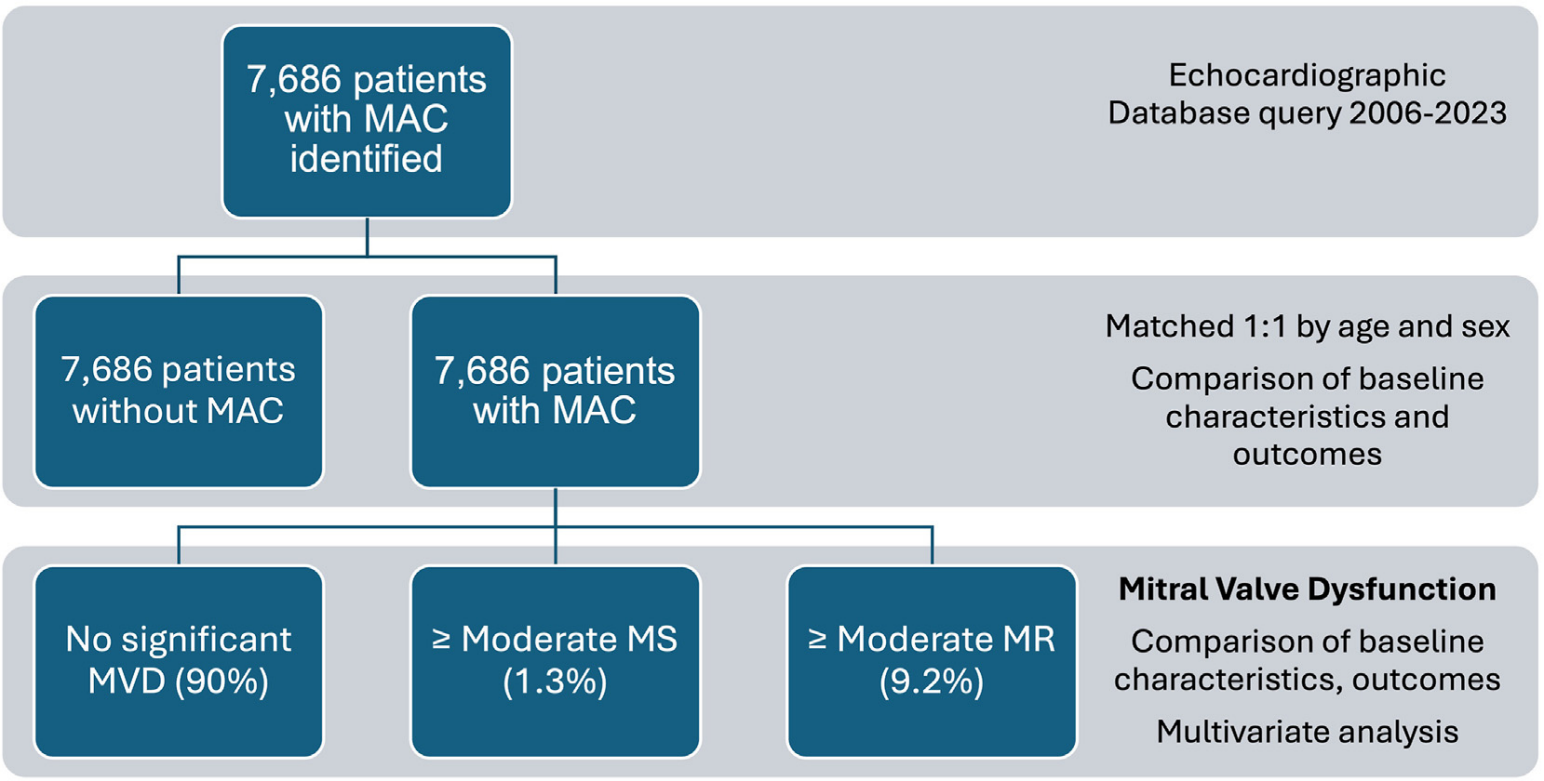
**Study chart describing the flow of patients in the study.** MAC, mitral annular calcification; MR, mitral regurgitation; MS, mitral stenosis; MVD, mitral valve disease.

We included 15,372 patients in the analysis: 7686 with MAC (median age, 76 years [68-84]; 58% women] and 7686 without MAC (median age, 77 years [69-84]; 58% women) (Figure 1). Baseline clinical characteristics of the study population are presented in Table 1. Patients with MAC had a higher burden of comorbidities, including diabetes, dyslipidemia, chronic obstructive pulmonary disease, hyperparathyroidism, and arterial hypertension. Patients with MAC also had worse renal function (median estimated glomerular filtration rate, 63 mL/min/1.73 m^2^ [45-80], vs 69 mL/min/1.73 m^2^ [52-83]; *P* < .001) and higher rates of dialysis (1.5% vs 0.3%; *P* < .001) compared with those without. In addition, patients with MAC had higher rates of atrial fibrillation (42% vs 34%; *P* < .001), coronary artery disease (50% vs 39%; *P* < .001), permanent pacemaker (14% vs 9%; *P* < .001), and transient ischemic attack/stroke (21% vs 19%; *P* < .001) compared with those without.

**Table 1:** Baseline characteristics of the study population.

Characteristics	Overall (N = 15,372)	No MAC (n = 7686)	MAC (n = 7686)	*P*
Age, y	77 (68-84)	77 (69-84)	76 (68-84)	NS
Female sex	8957 (58)	4466 (58)	4491 (58)	NS
White race	13,468 (89)	6609 (87)	6859 (91)	<.001
Smoking history	7344 (51)	3569 (49)	3775 (52)	.001
Diabetes	5630 (37)	2387 (31)	3243 (42)	<.001
Dyslipidemia	7389 (48)	3516 (46)	3873 (50)	<.001
eGFR, mL/min/1.73 m^2^	66 (48-82)	69 (52-83)	63 (45-80)	<.001
Dialysis	142 (0.9)	26 (0.3)	116 (1.5)	<.001
Cancer	3441 (22)	1759 (23)	1682 (22)	.136
COPD	2414 (16)	1040 (14)	1374 (18)	<.001
Hypothyroidism	3326 (22)	1531 (20)	1795 (23)	<.001
Hyperparathyroidism	752 (5)	280 (4)	472 (6)	<.001
Dementia	1127 (7)	628 (8)	499 (6)	<.001
Hypertension	13,090 (85)	6362 (83)	6728 (88)	<.001
Osteoporosis	2863 (19)	1441 (19)	1422 (19)	.694
Cirrhosis	290 (1.9)	109 (1.4)	181 (2.4)	<.001
Chronic kidney disease	4097 (27)	1668 (22)	2429 (32)	<.001
Atrial fibrillation	5827 (38)	2575 (34)	3252 (42)	<.001
Previous CABG	1150 (7)	417 (5)	733 (10)	<.001
Previous PCI	334 (2.2)	110 (1.4)	224 (2.9)	<.001
Coronary artery disease	6806 (44)	2998 (39)	3808 (50)	<.001
Previous MI	3300 (21)	1420 (18)	1880 (24)	<.001
Permanent pacemaker	1760 (11)	719 (9)	1041 (14)	<.001
Previous TIA/stroke	3092 (20)	1452 (19)	1640 (21)	<.001

Values are median (IQR) or n (%). Patients with MAC were matched 1:1 to patients without MAC.

CABG, coronary artery bypass graft; COPD, chronic obstructive pulmonary disease; eGFR, estimated glomerular filtration rate; MAC, mitral annular calcification; MI, myocardial infarction; PCI, percutaneous coronary intervention; TIA, transient ischemic attack.

### MAC and mitral valve dysfunction

Patients with MAC had higher rates of significant (≥moderate) mitral valve dysfunction (MVD). Of 7686 patients with MAC, 8.9% (n = 687) had ≥moderate MR (3.6%; *P* < .001), 1.1% (n = 81) had ≥moderate MS (0.02%; *P* < .001), and 0.26% (n = 20) had ≥moderate mixed mitral valve disease compared with those without. Clinical characteristics of patients with MAC and MVD are presented in Table 2. Patients with MAC and MS were more likely to be female and have diabetes, hyperparathyroidism, and chronic kidney disease relative to patients with MAC and MR. Patients with MAC and MR were older and more likely to have a previous MI when compared with patients with MAC and MS. Significant multivalvular heart disease was more frequently seen in patients with MAC compared with that in those without: ≥moderate aortic regurgitation (3% vs 0.3%; *P* < .001), ≥moderate aortic stenosis (9% vs 0.4%; *P* < .001), and moderate tricuspid regurgitation (12% vs 7%; *P* < .001).

**Table 2:** Clinical characteristics of patients with MAC and associated MVD.

	MAC and no significant MVD (n = 6877, 90%)	MAC and ≥moderate MS (n = 81, 1%)	MAC and ≥moderate MR (n = 687, 9%)	*P*
Age, y	76.3 (68.1-83.5)	76.7 (70.4-84.8)	82.2 (74.7-87.3)	<.001
Female sex	3982 (58)	56 (69)	434 (63)	.004
White race	6136 (91)	65 (82)	620 (92)	.013
Smoking history	3392 (52)	38 (50)	320 (49)	.288
Diabetes	2909 (42)	49 (60)	273 (40)	.002
Dyslipidemia	3444 (50)	37 (46)	365 (53)	.220
Hypertension	6009 (87)	68 (84)	615 (90)	.166
eGFR, mL/min/1.73 m^2^	64 (46-81)	45 (28-71)	52 (36-71)	<.001
Dialysis	97 (1)	2 (2)	16 (2)	.101
COPD	1210 (18)	17 (21)	142 (21)	.103
Hypothyroidism	1584 (23)	20 (25)	181 (26)	.141
Hyperparathyroidism	407 (6)	13 (16)	45 (7)	.001
Cirrhosis	159 (2)	5 (6)	15 (2)	.071
Chronic kidney disease	2081 (30)	41 (51)	287 (42)	<.001
Atrial fibrillation	2747 (40)	38 (47)	435 (63)	<.001
Previous CABG	630 (9)	7 (9)	91 (13)	.002
Previous MI	1605 (23)	23 (28)	240 (35)	<.001
Permanent pacemaker	840 (12)	18 (22)	163 (24)	<.001
Previous TIA/stroke	1441 (21)	19 (23)	163 (24)	.211
Echocardiographic variables				
≥Moderate AR	163 (3)	2 (4)	49 (8)	<.001
≥Moderate AS	509 (8)	18 (32)	97 (15)	<.001
≥Moderate TR	601 (9)	14 (17)	292 (43)	<.001
EF, %	57 (54-63)	62 (57-67)	53 (35-58)	<.001
LVEDD, mm	44 (40-49)	42 (36-47)	48 (42-54)	<.001
LVESD, mm	29 (25-34)	27 (24-32)	35 (28-43)	<.001
RVSP, mm Hg	32 (26-42)	43 (33-60)	42 (33-53)	<.001
TAPSE, mm	20 (17-24)	19 (16-22)	18 (14-22)	<.001
LA volume index	31 (24-40)	53 (38-66)	46 (34-56)	<.001

Values are median (IQR) or n (%). CABG, coronary artery bypass graft; COPD, chronic obstructive pulmonary disease; eGFR, estimated glomerular filtration rate; MAC, mitral annular calcification; MI, myocardial infarction; MR, mitral regurgitation; MS, mitral stenosis; MVD, mitral valve dysfunction; PCI, percutaneous coronary intervention; TIA, transient ischemic attack.

**Figure 2:**
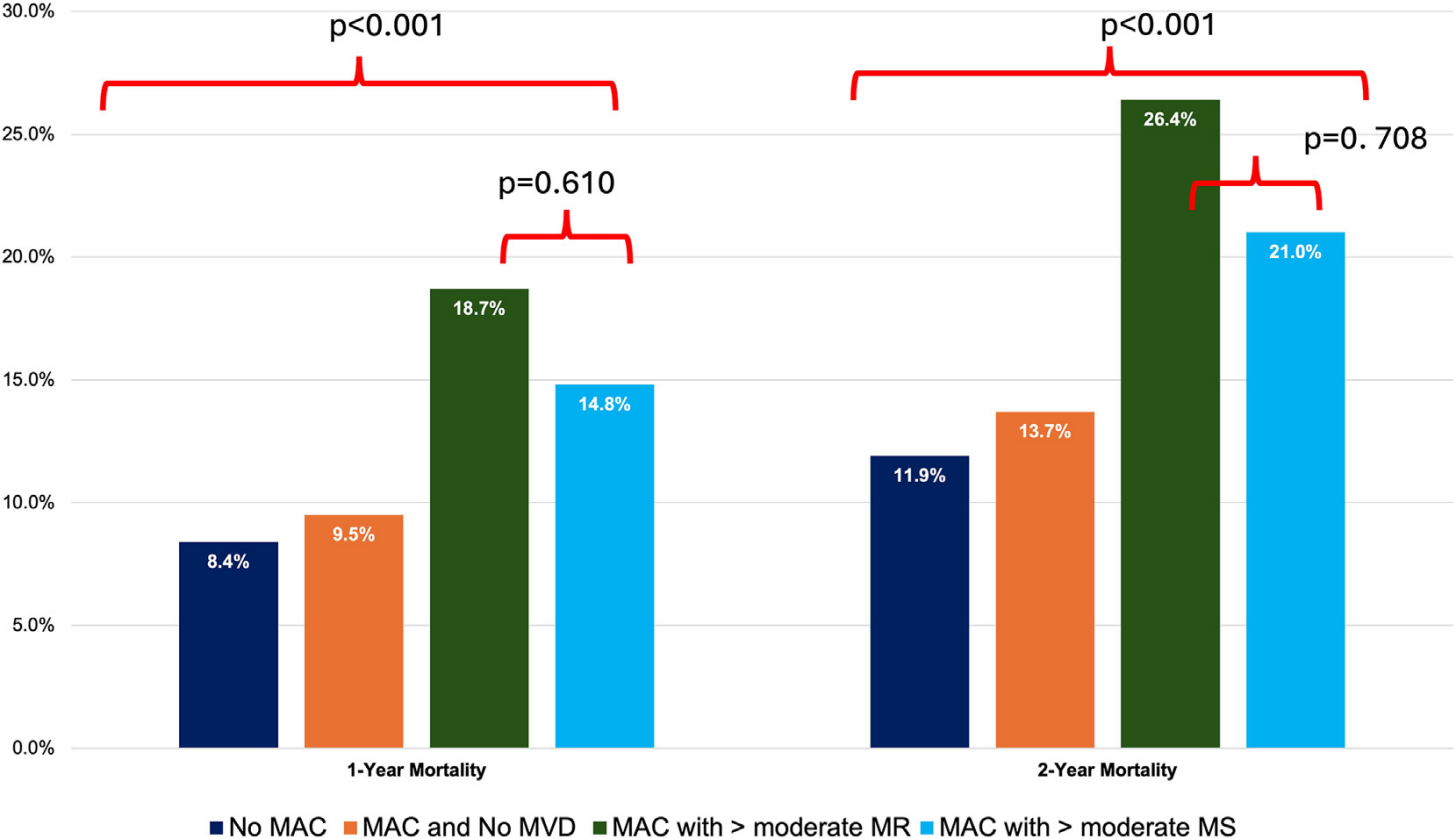
**Impact of mitral annular calcification (MAC) and associated mitral valve dysfunction (MVD) on mortality.** MR, mitral regurgitation; MS, mitral stenosis.

During a median follow-up time of 2.6 years (0.8-6.2), patients with MAC had higher overall mortality (28%) in comparison with patients without MAC (24%; *P* < .001). The presence of MVD significantly impacted mortality risk (Figure 2). Two-year mortality was 14%, 26%, and 21% for patients with MAC and no MVD, MAC with ≥moderate MR, and MAC with ≥moderate MS, respectively (*P* < .001). There were no differences in 1-year or 2-year mortality between patients with MAC and ≥moderate MR or ≥moderate MS (*P* = .610 and .708, respectively). Multivariate predictors of mortality for patients with MAC are presented in Table 3. These include medical comorbidities, evidence of multivalvular disease, and left ventricular chamber dilation/dysfunction. CV-related mortality was 26% in patients with moderate-severe/severe MS and/or MR who did not receive an intervention and 19% in those who did (*P* = .180).

**Table 3:** Factors associated with mortality in patients with MAC.

Variable	Model 1 (univariate)		Model 2 (age + sex)		Model 3 (backward stepwise)	
	HR (95% CI)	*P*	HR (95% CI)	*P*	HR (95% CI)	*P*
Male sex	1.075 (0.957-1.209)	.222	—	—	1.136 (0.984-1.313)	.082
Age, per 10 y	1.658 (1.560-1.762)	<.001	—	—	1.677 (1.556-1.809)	<.001
Diabetes	1.166 (1.039-1.310)	.009	1.332 (1.185-1.498)	<.001	1.286 (1.115-1.483)	.001
Dyslipidemia	0.706 (0.628-0.792)	<.001	0.707 (0.630-0.795)	<.001	0.661 (0.577-0.757)	<.001
Dialysis	1.355 (0.911-2.014)	.133	1.867 (1.254-2.777)	.002		
Cancer	1.261 (1.107-1.435)^a^	<.001	1.082 (0.949-1.233)	.239		
Chronic obstructive pulmonary disease	1.490 (1.305-1.702)	<.001	1.543 (1.351-1.763)	<.001	1.499 (1.281-1.755)	<.001
Hypothyroid	1.046 (0.916-1.194)	.509	1.008 (0.881-1.154)	.904		
Hyperparathyroidism	1.107 (0.885-1.386)	.374	1.215 (0.970-1.521)	.09		
Dementia	1.231 (0.999-1.518)	.051	0.960 (0.777-1.184)	.7		
Hypertension	1.158 (0.944-1.420)	.16	1.028 (0.838-1.261)	.793		
Osteoporosis	0.968 (0.836-1.121)	.662	0.858 (0.735-1.002)	.054		
Cirrhosis	2.241 (1.706-2.946)^a^	<.001	2.868 (2.178-3.776)	<.001	2.357 (1.697-3.275)	<.001
Chronic kidney disease	1.379 (1.226-1.552)	<.001	1.304 (1.159-1.468)	<.001	1.138 (0.984-1.315)	.081
Coronary artery disease	1.052 (0.937-1.181)^a^	.392	1.030 (0.914-1.159)	.63		
Atrial fibrillation	1.539 (1.370-1.728)^a^	<.001	1.264 (1.124-1.422)	<.001		
Percutaneous coronary intervention	0.848 (0.597-1.205)	.359	0.936 (0.658-1.332)	.715		
Myocardial infarction	1.371 (1.212-1.552)	<.001	1.343 (1.186-1.521)	<.001		
Permanent pacemaker	1.281 (1.103-1.489)	.001	1.150 (0.988-1.338)	.072		
Transient ischemic attack/stroke	1.081 (0.944-1.237)	.26	0.999 (0.873-1.144)	.99		
≥Moderate mitral stenosis	1.498 (0.962-2.330)	.073	1.361 (0.874-2.118)	.172		
≥Moderate mitral regurgitation	1.977 (1.689-2.315)	<.001	1.605 (1.368-1.882)	<.001	1.230 (1.006-1.504)	.044
≥Moderate aortic stenosis	1.704 (1.427-2.034)	<.001	1.364 (1.141-1.632)	.001	1.223 (0.985-1.517)	.068
≥Moderate aortic regurgitation	1.456 (1.077-1.968)	.015	1.127 (0.832-1.525)	.441		
≥Moderate tricuspid regurgitation	2.250 (1.954-2.591)	<.001	1.795 (1.554-2.073)	<.001	1.486 (1.244-1.773)	<.001
Left ventricular ejection fraction, 10% increase	0.770 (0.737-0.805)	<.001	0.781 (0.746-0.817)	<.001	0.776 (0.730-0.824)	<.001
Left ventricular end-diastolic dimension, mm, 10.0 mm	0.982 (0.902-1.070)	.684	1.116 (1.021-1.220)	.016	0.871 (0.788-0.963)	.007
Left ventricular end-systolic dimension, mm, 10.0 mm	1.223 (1.137-1.316)	<.001	1.281 (1.187-1.382)	<.001		

MAC, mitral annular calcification.

a Variable violates proportional-hazards assumption in a univariate analysis.

### MAC computed tomography score and mitral valve interventions

**Figure 3:**
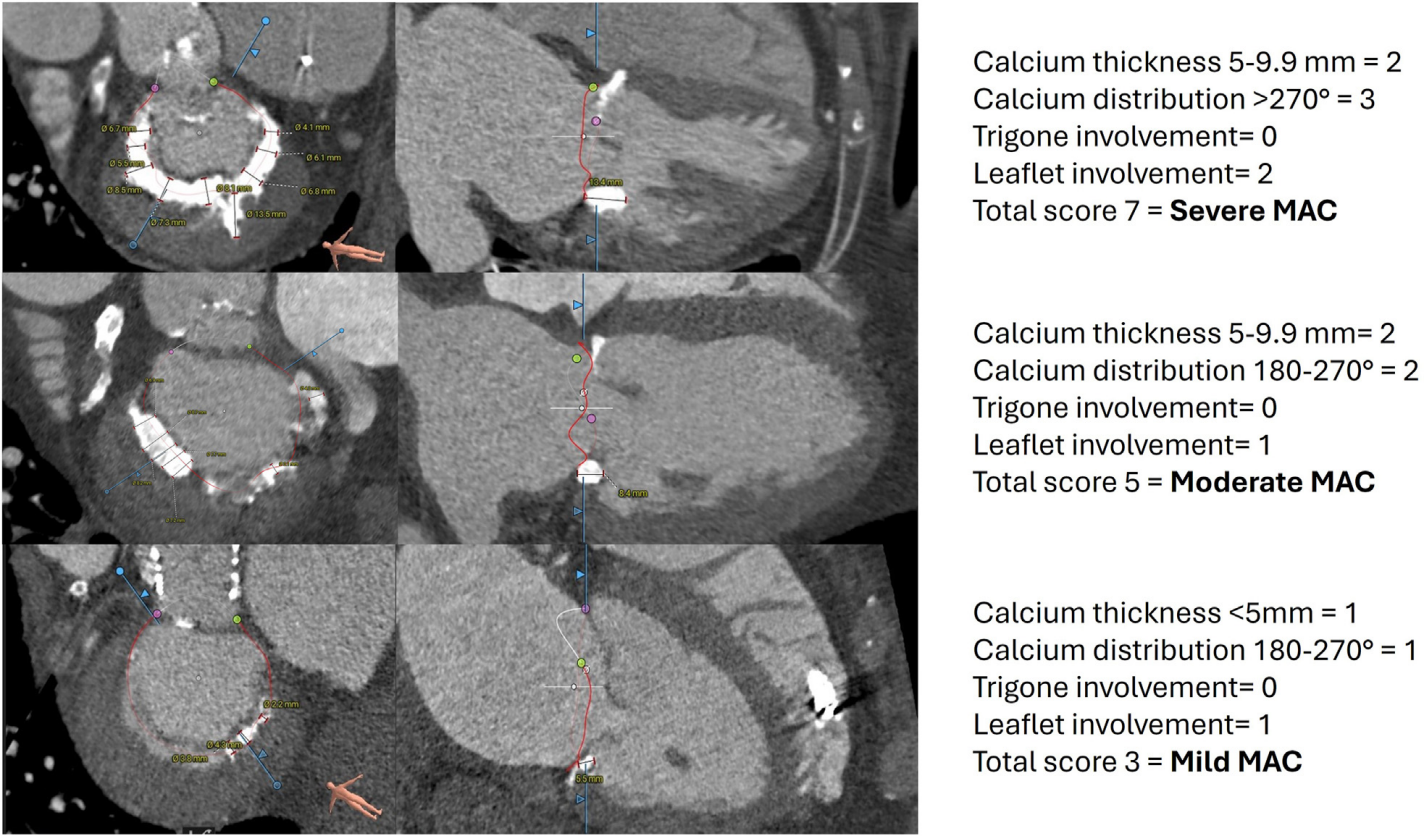
**Case examples of patients with mild, moderate, and severe mitral annular calcification (MAC) calcium score**.

Cardiac computed tomography studies were available for interpretation and MAC score calculation in 134 patients. Sixty percentage of patients had a calcium thickness <5.0 mm, 75% had a calcium distribution <180°, 88% had no involvement of the mitral valve trigones, and 44% had 1 leaflet involved. The median MAC score was 4 (3-5) and 38% had mild, 53% moderate, and 9% severe MAC calcium scores (Figure 3; Supplemental Table S1).

Surgical or transcatheter mitral valve interventions in patients with MAC and significant MVD were infrequently performed (n = 136/788, 17.2%). The type and frequency of mitral valve interventions and concomitant procedures performed are presented in Supplemental Table S2. The most common reasons for not offering an intervention were as follows: (1) patients were deemed high risk for surgery and had no transcatheter option (38%), (2) patients with stable MR/MS with medical treatment (35%), and (3) patients had multiple comorbidities and were ultimately enrolled in hospice/palliative care (27%).

### MAC and MR severity

Of 3732 patients with MAC and MR, 3025 had mild/mild-moderate MR (81%), 501 moderate MR (13.4%), and 206 moderate-severe/severe (5.5%) MR. Clinical characteristics of patients with MAC and MR are presented in (Supplemental Table S3). Patients with severe MR were older (median age, 82 years [76-87]), had worse renal function (estimated glomerular filtration rate, 56 mL/min/1.73 m^2^ [33-74]), had a higher prevalence of hypothyroidism (34%), and had higher rates of CV disease such as atrial fibrillation (67%), coronary artery bypass graft (16%), and permanent pacemakers (25%) in comparison with those patients with patients with lesser degrees of MR.

After a median follow-up time of 2.6 years (0.9-5.5), patients with moderate-severe/severe MR had significantly higher mortality (49%) compared with patients with mild/mild-moderate MR (32%; *P* < .001) (Supplemental Figure S1). After adjusting for confounding variables, factors associated with mortality risk included older age (hazard ratio [HR], 1.604; 95% CI, 1.452-1.773; *P* < .001), diabetes (HR, 1.324; 95% CI, 1.101-1.592; *P* < .003), chronic obstructive pulmonary disease (HR, 1.458; 95% CI, 1.191-1.784; *P* < .001), cirrhosis (HR, 1.866; 95% CI, 1.189-2.929; *P* = .007), degree of MR (compared with mild; moderate MR—HR, 1.335; 95% CI, 1.065-1.673; *P* = .012; severe MR—HR, 1.783; 95% CI, 1.292-2.460; *P* < .001), and left ventricular ejection fraction (increase in 10 units; HR, 0.758; 95% CI, 0.704-0.816; *P* < .001) (Supplemental Table S4).

## Discussion

We conducted a large, observational study of patients with MAC to compare clinical characteristics and outcomes relative to patients without MAC matched by age and sex. We also assessed the impact of MVD, including regurgitation and stenosis, among patients with MAC. Several key findings are noted (Central Illustration). First, patients with MAC have a high burden of cardiac and noncardiac comorbidities relative to patients without MAC. Second, 1 in 10 patients with MAC has significant MVD, with MR being significantly more common than MS. Third, the type and severity of MVD are independently associated with mortality, which is highest for patients with MAC and ≥moderate MR (26% at 2 years) vs patients with MAC without MVD (14% at 2 years). Fourth, patients with MS and MR showed differences in baseline comorbidities and CV risk factors: MS was associated with conditions that cause dysregulation in calcium and phosphorus homeostasis (chronic kidney disease, hyperparathyroidism, and diabetes), whereas MR was more commonly associated with ischemic heart disease and atrial fibrillation. Finally, transcatheter and surgical interventions were infrequently performed in clinical practice (17.2% of patients with MAC and significant MVD). Reasons for treatment denial included high surgical risk coupled with no transcatheter options available in one-third of patients and clinical stabilization of valvular heart disease with medical management alone.

**Central Illustration:**
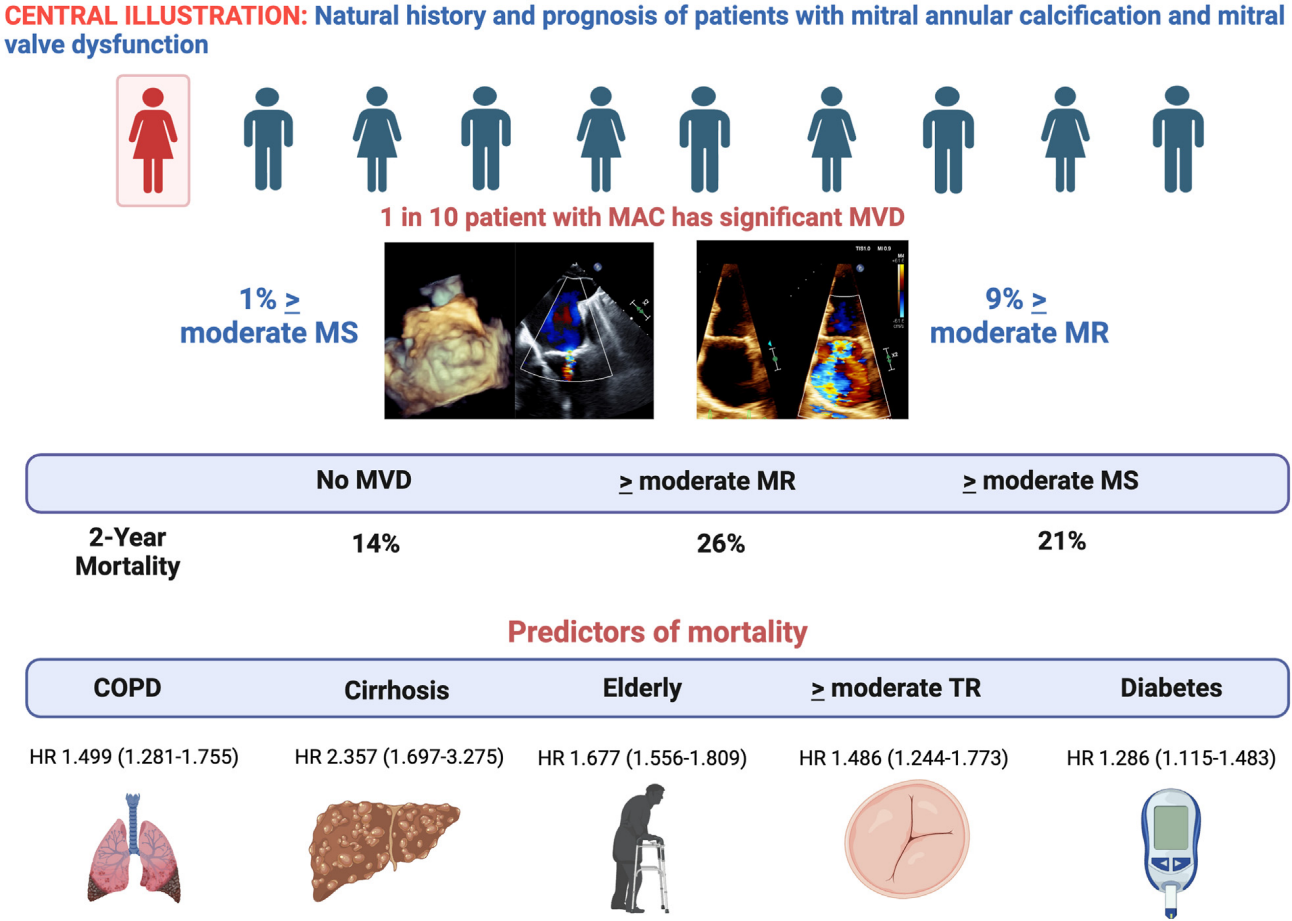
**Natural history and prognosis of patients with mitral annular calcification and mitral valve dysfunction.** COPD, chronic obstructive pulmonary disease; HR, hazard ratio; MAC, mitral annular calcification; MR, mitral regurgitation; MS, mitral stenosis; MVD, mitral valve dysfunction; TR, tricuspid regurgitation. Created in https://BioRender.com

The prevalence of MAC varies widely between studies, which can be explained by the age and comorbidities of the studied population as well as the diagnostic methods used to confirm the diagnosis of MAC. MAC is more frequently found in the elderly population, women, and patients with chronic kidney disease and increases the risk of significant MVD.^3^^,^^12^^,^^13^ Ten percentage of patients with MAC develop significant MVD as shown by our study and others, MR being the predominant form of MVD in this population.^4^^,^^13^ In addition, MS was more commonly seen in patients with a higher prevalence of comorbidities that are associated with calcium and phosphorus dysregulation, such as chronic kidney disease and hyperparathyroidism, which increases the risk of developing MAC by 4-fold.^14^^,^^15^

The presence of MAC was also associated with multivalvular disease, including a higher prevalence of significant aortic valve disease (stenosis and regurgitation) and tricuspid regurgitation. MAC is commonly seen in patients with severe aortic stenosis.^16^, ^17^, ^18^ In a meta-analysis of 5822 patients undergoing transcatheter aortic valve replacement, ∼40% of patients had evidence of MAC, 6% of them had MVD, and the presence of MAC and MVD was associated with an increased 1-year mortality.^19^ Similarly, ∼14% of patients with tricuspid regurgitation have some degree of MAC, which can cause tricuspid regurgitation from increased pulmonary pressures and tricuspid annular dilation rather than direct leaflet involvement.^20^^,^^21^

As seen in other series, MAC significantly increases overall mortality.^1^^,^^4^^,^^22^ Our study highlights that in patients with MAC, the presence and severity of MVD appear to be the main driver of mortality. The 2-year mortality for patients with MAC without MVD (13.7%) is slightly higher than the mortality of patients without MAC (11.9%) matched by age and sex. However, the risk of mortality in patients with MAC increases significantly if there is evidence of clinically significant MR (26.4%) or MS (21.0%).^3^ Other predictors of mortality in patients with MAC included renal, pulmonary, and structural heart conditions.

Treatment options for patients with MAC and MVD are limited due to several factors.^7^^,^^23^ Patients with MAC are frail and carry a high burden of comorbidities, increasing the overall risk of surgery. In addition, technical challenges exist with surgical and transcatheter approaches, limiting the pool of patients that can benefit from treatment. Mitral valve repair has been described as the preferred treatment strategy in patients with mild MAC and otherwise repairable anatomies.^24^ In patients who are not candidates for repair, surgical replacement should be considered with a plan to either avoid annular decalcification (respect) or decalcification with annular reconstruction (resect) of the mitral annulus as the main surgical dilemma. Irrespective of the surgical strategy, MAC is associated with increased operative mortality and postoperative complications in patients undergoing surgical mitral valve replacement.^25^, ^26^, ^27^

Mitral transcatheter edge-to-edge repair has been performed in selected patients with MAC. Intermediate-term mortality and symptom improvement appear to be worse in patients with severe MAC.^28^ Finally, transcatheter mitral valve replacement strategies and devices for patients with MAC are currently under investigation. Complications and procedural challenges seen with these devices have slowed the progress and adoption of this treatment strategy.^29^ The MITRAL trial^30^ evaluated transcatheter mitral valve replacement in patients with MAC using commercially available balloon-expandable aortic stenosis platforms (SAPIEN XT and SAPIEN 3; Edwards Lifesciences). Patients had a sustained improvement in symptoms and quality of life that peaked early after the procedure without significant changes in the long-term follow-up. However, the survival rate was only 33% during a 5-year follow-up period, which can be attributed to the high number of comorbidities frequently seen in patients with MAC and the procedural success rates described in the study (53%). Finally, transcatheter mitral valve replacement with dedicated mitral devices is an emerging field where new platforms and devices to address the described treatment challenges are being developed.^31^ The Feasibility Study of the Tendyne Mitral Valve System in Mitral Annular Calcification (NCT03539458) reported 40% mortality at 1 year.^32^ In our study, <20% of patients had an intervention performed, highlighting the importance of addressing this unmet clinical need.

### Limitations

This study has several strengths, including a large sample size and midterm follow-up. However, our study has intrinsic limitations common to observational, retrospective, and single-center studies, including unmeasured confounders and lack of an independent events committee for adjudication of clinical outcomes. It should be noted that the primary outcome of the study, overall mortality, is less subject to ascertainment bias. The echocardiograms included in the study were evenly distributed between inpatient (n = 3792, 49.2%) and outpatient (n = 3911, 50.8%) settings. Loading conditions during acute hospitalization could have impacted the severity of MS and MR observed in the echocardiograms. We attempted to calculate the severity of MAC by computed tomography analysis. However, the studies were done predominantly in patients with severe aortic stenosis undergoing preprocedural planning for transcatheter aortic valve replacement at our institution. Therefore, the sample size for comparisons is small, and the report of computed tomography data are mainly descriptive. Finally, additional outcomes, such as heart failure admissions, quality of life, and long-term follow-up beyond 3 years in patients with MAC, were not available.

## Conclusions

Mitral annular calcification is a complex disease commonly seen in frail and highly comorbid patients. Ten percentage of patients with MAC have clinically significant (≥moderate) mitral valve dysfunction, with MR being more common than MS. The presence of MVD is associated with significantly increased mortality in patients with MAC.

## References

[1] Fox C.S., Vasan R.S., Parise H. (2003). Mitral annular calcification predicts cardiovascular morbidity and mortality: the Framingham Heart Study. Circulation.

[2] Barasch E., Gottdiener J.S., Larsen E.K., Chaves P.H., Newman A.B., Manolio T.A. (2006). Clinical significance of calcification of the fibrous skeleton of the heart and aortosclerosis in community dwelling elderly. The Cardiovascular Health Study (CHS). Am Heart J.

[3] Churchill T.W., Yucel E., Deferm S., Levine R.A., Hung J., Bertrand P.B. (2022). Mitral valve dysfunction in patients with annular calcification: JACC review topic of the week. J Am Coll Cardiol.

[4] Kato N., Guerrero M., Padang R. (2022). Prevalence and natural history of mitral annulus calcification and related valve dysfunction. Mayo Clin Proc.

[5] Museedi A.S., Le Jemtel T.H. (2024). Mitral annular calcification-related valvular disease: a challenging entity. J Clin Med.

[6] Elmariah S., Budoff M.J., Delaney J.A. (2013). Risk factors associated with the incidence and progression of mitral annulus calcification: the multi-ethnic study of atherosclerosis. Am Heart J.

[7] Chehab O., Roberts-Thomson R., Bivona A. (2022). Management of patients with severe mitral annular calcification: JACC state-of-the-art review. J Am Coll Cardiol.

[8] Zoghbi W.A., Adams D., Bonow R.O. (2017). Recommendations for noninvasive evaluation of native valvular regurgitation: a report from the American Society of Echocardiography developed in collaboration with the Society for Cardiovascular Magnetic Resonance. J Am Soc Echocardiogr.

[9] Baumgartner H., Hung J., Bermejo J. (2017). Recommendations on the echocardiographic assessment of aortic valve stenosis: a focused update from the European Association of Cardiovascular Imaging and the American Society of Echocardiography. J Am Soc Echocardiogr.

[10] Baumgartner H., Hung J., Bermejo J. (2009). Echocardiographic assessment of valve stenosis: EAE/ASE recommendations for clinical practice. J Am Soc Echocardiogr.

[11] Guerrero M., Wang D.D., Pursnani A. (2020). A cardiac computed tomography-based score to categorize mitral annular calcification severity and predict valve embolization. JACC Cardiovasc Imaging.

[12] Okura H., Nakada Y., Nogi M. (2021). Prevalence of mitral annular calcification and its association with mitral valvular disease. Echocardiography.

[13] Dal-Bianco J.P., Levine R.A., Hung J. (2024). Mitral annular calcification and valve dysfunction: insights and future directions. J Am Soc Echocardiogr.

[14] Abd Alamir M., Radulescu V., Goyfman M. (2015). Prevalence and correlates of mitral annular calcification in adults with chronic kidney disease: results from CRIC study. Atherosclerosis.

[15] Kipourou K., O’Driscoll J.M., Sharma R. (2022). Valvular heart disease in patients with chronic kidney disease. Eur Cardiol.

[16] Abramowitz Y., Kazuno Y., Chakravarty T. (2017). Concomitant mitral annular calcification and severe aortic stenosis: prevalence, characteristics and outcome following transcatheter aortic valve replacement. Eur Heart J.

[17] Thanassoulis G., Campbell C.Y., Owens D.S. (2013). Genetic associations with valvular calcification and aortic stenosis. N Engl J Med.

[18] Jassal D.S., Tam J.W., Bhagirath K.M. (2008). Association of mitral annular calcification and aortic valve morphology: a substudy of the aortic stenosis progression observation measuring effects of rosuvastatin (ASTRONOMER) study. Eur Heart J.

[19] Ahmad S., Yousaf A., Ghumman G.M. (2024). Outcomes of transcatheter aortic valve replacement in patients with mitral annular calcification and concomitant mitral valve dysfunction: a systematic review and meta-analysis. Cardiovasc Revasc Med.

[20] Movahed M.R., Saito Y., Ahmadi-Kashani M., Ebrahimi R. (2007). Mitral annulus calcification is associated with valvular and cardiac structural abnormalities. Cardiovasc Ultrasound.

[21] Al-Abcha A., Abbasi M., El-Am E. (2024). Staging extramitral cardiac damage in mitral annular calcification with mitral valve dysfunction. JACC Cardiovasc Interv.

[22] Lee H.J., Seo J., Gwak S.Y. (2023). Risk factors and outcomes with progressive mitral annular calcification. J Am Heart Assoc.

[23] Guerrero M.E., Grayburn P., Smith R.L.I.I. (2023). Diagnosis, classification, and management strategies for mitral annular calcification: a heart valve collaboratory position statement. JACC Cardiovasc Interv.

[24] Bedeir K., Kaneko T., Aranki S. (2019). Current and evolving strategies in the management of severe mitral annular calcification. J Thorac Cardiovasc Surg.

[25] Alexis S.L., Malik A.H., El-Eshmawi A. (2021). Surgical and transcatheter mitral valve replacement in mitral annular calcification: a systematic review. J Am Heart Assoc.

[26] Uchimuro T., Fukui T., Shimizu A., Takanashi S. (2016). Mitral valve surgery in patients with severe mitral annular calcification. Ann Thorac Surg.

[27] Kaneko T., Hirji S., Percy E. (2019). Characterizing risks associated with mitral annular calcification in mitral valve replacement. Ann Thorac Surg.

[28] Hatab T., Bou Chaaya R.G., Zaid S. (2023). Feasibility and outcomes of mitral transcatheter edge-to-edge repair in patients with variable degrees of mitral annular calcification. J Am Heart Assoc.

[29] Hasan S.A., Morsi M., Frakes B.S. (2024). Management strategies and prognosis of patients ineligible for transcatheter mitral valve replacement. Cardiovasc Revasc Med.

[30] Guerrero M.E., Eleid M.F., Wang D.D. (2023). 5-Year prospective evaluation of mitral valve-in-valve, valve-in-ring, and valve-in-MAC outcomes: MITRAL trial final results. JACC Cardiovasc Interv.

[31] Kietrsunthorn P.S., Ghrair F., Schelegle A.R., Foerst J.R. (2024). Transcatheter mitral valve therapies in patients with mitral annular calcification. Interv Cardiol Clin.

[32] Gossl M., Thourani V., Babaliaros V. (2022). Early outcomes of transcatheter mitral valve replacement with the Tendyne system in severe mitral annular calcification. EuroIntervention.

