# Supplementary material for: Mitral Annular Calcification: Natural History, Prognosis, and Clinical Outcomes
Source: J Soc Cardiovasc Angiogr Interv. 2025 Jul 22;4(8):103732. doi: 10.1016/j.jscai.2025.103732 (PMC12462118; doi:10.1016/j.jscai.2025.103732)
Supplement: Supplemental Tables and Figure [file mmc1.docx]

**SUPPLEMENTAL MATERIAL**

**Supplemental Table S1.** MAC computed tomography score.

| **MAC Score** | N=134 |
| --- | --- |
| Calcium Thickness Average, mean ± SD | 4.85 ± 2.51 |
| Calcium Thickness  <5  5-10  >10 | 80 (60)  51 (38)  3 (2) |
| **Calcium Distribution**  <180  180-270  >270 | 101 (75)  22 (16)  11 (8) |
| **Trigone Involvement**  None  1  2 | 118 (88)  15 (11)  1 (1) |
| **Leaflet Involvement**  None  1  2 | 21 (16)  59 (44)  54 (40) |
| **MAC grade severity**  Mild  Moderate  Severe | 51 (38)  71 (53)  12 (9) |
| **Total Score,** median (IQR) | 4 (3, 5) |
| **Calcium Volume**, median (IQR) | 923 (368, 2269) |

**Supplemental Table S2.** Type and frequency of mitral valve interventions.

| **MV Intervention** | N=136 | Concomitant procedures in patients treated with surgery |
| --- | --- | --- |
| M-TE ER  TMVR  sMVR  sMVr | 28 (21)  2 (1)  48 (35)  58 (43) | LAAC (25)  SAVR (13)  sTVr (12)  CABG (11)  MAZE (3) |

CABG: coronary artery bypass graft; LAAC: left atrial appendage clippage; SAVR: surgical aortic valve replacement; M-TEER: mitral transcatheter edge-to-edge repair; sMVR: surgical mitral valve replacement; sMVr: surgical mitral valve repair; sTVr: surgical tricuspid valve repair; TMVR: transcatheter mitral valve replacement

**Supplemental Table S3**. Clinical characteristics of patients with MAC and Various Degrees of Mitral Regurgitation Severity.

|  | **Mild/Mild-Moderate**  **(N=3025)** | **Moderate**  **(N=501)** | **Moderate-Severe/Severe**  **(N=206)** | **p-value** |
| --- | --- | --- | --- | --- |
| Age, median (IQR) | 78.7 (71.1, 85.3) | 82.0 (74.6, 86.9) | 82.4 (76.3, 87.6) | <0.001 |
| Female, n (%) | 1849 (61) | 314 (63) | 136 (66) | 0.327 |
| White race, n (%) | 2723 (92) | 456 (93) | 182 (90) | 0.505 |
| Smoking Hx, n (%) | 1459 (51) | 230 (49) | 103 (53) | 0.511 |
| Diabetes, n (%) | 1207 (40) | 191 (38) | 89 (43) | 0.451 |
| Dyslipidema, n (%) | 1534 (51) | 268 (53) | 110 (53) | 0.419 |
| eGFR, median (IQR) | 61 (44, 79) | 51 (36, 70) | 56 (33, 74) | <0.001 |
| Dialysis, n (%) | 51 (2) | 10 (2) | 6 (3) | 0.411 |
| Cancer, n (%) | 698 (23) | 121 (24) | 56 (27) | 0.372 |
| COPD, n (%) | 509 (17) | 104 (21) | 44 (21) | 0.035 |
| Hypothyroidism, n (%) | 734 (24) | 116 (23) | 71 (34) | 0.003 |
| Hyperparathyroidism, n (%) | 195 (6) | 32 (6) | 16 (7) | 0.891 |
| Dementia, n (%) | 190 (6) | 38 (8) | 16 (8) | 0.420 |
| Hypertension, n (%) | 2679 (89) | 448 (89) | 186 (90) | 0.663 |
| Osteoporosis, n (%) | 605 (20) | 98 (20) | 38 (18) | 0.850 |
| Cirrhosis, n (%) | 74 (2) | 10 (2) | 5 (2) | 0.829 |
| CKD, n (%) | 1018 (34) | 201 (40) | 97 (47) | <0.001 |
| Atrial Fibrillation, n (%) | 1462 (48) | 312 (62) | 137 (67) | <0.001 |
| CABG, n (%) | 312 (10) | 60 (12) | 33 (16) | 0.027 |
| PCI, n (%) | 91 (3) | 27 (5) | 6 (3) | 0.021 |
| CAD, n (%) | 1569 (52) | 288 (57) | 131 (64) | 0.001 |
| MI, n (%) | 793 (26) | 178 (36) | 70 (34) | <0.001 |
| Permanent Pacemaker, n (%) | 448 (15) | 119 (24) | 52 (25) | <0.001 |
| TIA/Stroke, n (%) | 668 (22) | 119 (24) | 52 (25) | 0.440 |
| Death, n (%) | 958 (32) | 226 (45) | 100 (49) | <0.001 |
| Death at 1-Year, n (%) | 327 (11) | 84 (17) | 45 (22) | <0.001 |
| Death at 2-years, n (%) | 482 (16) | 124 (25) | 60 (29) | <0.001 |

CABG: coronary artery bypass graft; CAD: coronary artery disease; CKD: chronic kidney disease; COPD: chronic obstructive pulmonary disease; COVID-19: Coronavirus disease; eGFR: estimates glomerular filtration rate; MI: myocardial infarction; PCI: percutaneous coronary intervention; TIA: transient ischemic attack

**Supplemental Table S4**. Factors associated with mortality in patients with MAC and MR.

|  | **Univariate Model Hazard Ratio** | **P-value** | **Partly Adjusted Model Hazard Ratio *** | **p-value** | **Fully Adjusted Model Hazard Ratio^** | **p-value** |
| --- | --- | --- | --- | --- | --- | --- |
| Age (10-years) | 1.571 [1.444, 1.708] | <0.001 | --- | --- | 1.604 [1.452, 1.773] | <0.001 |
| Male Sex | 1.072 [0.918, 1.251] | 0.381 | --- | --- | 1.075 [0.893, 1.295] | 0.444 |
| White race | 0.960 [0.721, 1.278] | 0.780 | 0.829 [0.623, 1.105] | 0.201 |  |  |
| Smoking History | 1.114 [0.953, 1.303] | 0.174 | 1.197 [1.018, 1.407] | 0.029 |  |  |
| Diabetes | 1.268 [1.088, 1.476] | 0.002 | 1.481 [1.268, 1.730] | <0.001 | 1.324 [1.101, 1.592] | 0.003 |
| Dyslipidema | 0.670 [0.575, 0.781] | <0.001 | 0.683 [0.586, 0.796] | <0.001 | 0.664 [0.558, 0.791] | <0.001 |
| Dialysis | 1.114 [0.656, 1.892] | 0.689 | 1.425 [0.838, 2.424] | 0.191 |  |  |
| Cancer | 1.071 [0.901, 1.273] | 0.439 | 0.971 [0.816, 1.156] | 0.744 |  |  |
| COPD | 1.479 [1.239, 1.764] | <0.001 | 1.532 [1.283, 1.830] | <0.001 | 1.458 [1.191, 1.784] | <0.001 |
| Hypothyroidism | 1.058 [0.891, 1.256] | 0.519 | 1.035 [0.869, 1.232] | 0.703 |  |  |
| Hyperparathyroidism | 1.095 [0.821, 1.461] | 0.536 | 1.190 [0.892, 1.588] | 0.238 |  |  |
| Dementia | 1.133 [0.854, 1.505] | 0.384 | 0.958 [0.720, 1.272] | 0.765 |  |  |
| Hypertension | 1.142 [0.863, 1.512]* | 0.353 | 1.084 [0.818, 1.435] | 0.576 |  |  |
| Osteoporosis | 0.993 [0.823, 1.197] | 0.938 | 0.966 [0.792, 1.177] | 0.728 |  |  |
| Cirrhosis | 1.485 [0.980, 2.25] | 0.062 | 1.865 [1.227, 2.834] | 0.004 | 1.866 [1.189, 2.929] | 0.007 |
| CKD | 1.284 [1.101, 1.498] | 0.001 | 1.225 [1.050, 1.430] | 0.010 | 1.192 [0.992, 1.432] | 0.061 |
| Atrial Fibrillation | 1.287 [1.103, 1.502]* | 0.001 | 1.066 [0.911, 1.247] | 0.428 |  |  |
| CABG | 1.188 [0.949, 1.486] | 0.133 | 1.153 [0.917, 1.450] | 0.222 |  |  |
| PCI | 0.882 [0.577, 1.349] | 0.563 | 0.947 [0.618, 1.452] | 0.802 |  |  |
| CAD | 0.956 [0.820, 1.113] | 0.561 | 0.956 [0.817, 1.120] | 0.580 |  |  |
| MI | 1.337 [1.140, 1.568] | <0.001 | 1.326 [1.129, 1.558] | 0.001 |  |  |
| Permanent Pacemaker | 1.214 [1.006, 1.464] | 0.043 | 1.105 [0.913, 1.337] | 0.305 |  |  |
| TIA/Stroke | 0.985 [0.823, 1.178] | 0.866 | 0.957 [0.800, 1.144] | 0.627 |  |  |
| MR Severity (Mild is Ref)  Moderate  Severe | 1.63 [1.34, 1.99]  2.01 [1.54, 2.63] | <0.001  <0.001 | ---  --- | ---  --- | 1.335 [1.065, 1.673]  1.783 [1.292, 2.460] | 0.012  <0.001 |
| Left Ventricular ejection fraction (10%) | 0.776 [0.736, 0.819] | <0.001 | 0.784 [0.740, 0.830] | <0.001 | 0.758 [0.704, 0.816] | <0.001 |
| Left ventricular end-diastolic dimension mm (10) | 1.031 [0.927, 1.147] | 0.575 | 1.109 [0.988, 1.244] | 0.080 | 0.879 [0.773, 1.000] | 0.049 |
| Left ventricular end-systolic dimension mm (10) | 1.226 [1.125, 1.336] | <0.001 | 1.266 [1.151, 1.394] | <0.001 |  |  |

* Partly adjusted model is adjusted only for age, sex, and MR severity

^ Fully adjusted model adjusts for age, sex, and MR severity and performed a backward stepwise selection

CABG: coronary artery bypass graft; CAD: coronary artery disease; CKD: chronic kidney disease; COPD: chronic obstructive pulmonary disease; COVID-19: Coronavirus disease; eGFR: estimates glomerular filtration rate; MI: myocardial infarction; MR: mitral regurgitation; PCI: percutaneous coronary intervention; TIA: transient ischemic attack

**Supplemental Figures**

**Supplemental Figure S1.** Kaplan-Meier survival curves for patients with MAC and mitral regurgitation (MR) according to MR grade.

**
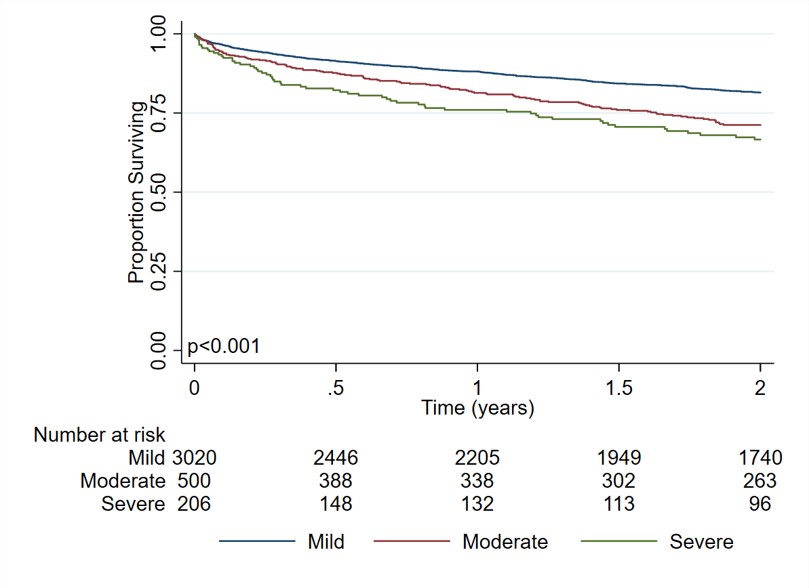
**
